# AI Adoption in US Cancer Centers: National Cross-Sectional Study of Institutional and Policy Determinants

**DOI:** 10.2196/96231

**Published:** 2026-08-26

**Authors:** Jingjing Gao, Muinat Abolore Idris, Eric C Jones, Louis D Brown, Jack Tsai

**Affiliations:** 1 Department of Management, Policy and Community Health, School of Public Health, The University of Texas Health Science Center at Houston, 7000 Fannin Street, Houston, TX, 77225, United States, 1 9802136680; 2 Department of Environmental & Occupational Health Sciences, School of Public Health, The University of Texas Health Science Center at Houston, Houston, TX, United States; 3 School of Public Health, The University of Texas Health Science Center at Houston, Houston, TX, United States; 4 Department of Psychiatry, Yale University, New Haven, CT, United States

**Keywords:** artificial intelligence, cancer care, health technology adoption, innovation diffusion, health equity

## Abstract

**Background:**

AI is increasingly being integrated into cancer screening, treatment, and patient care. However, AI adoption across cancer centers varies, raising concerns about unequal access to AI-enabled cancer care.

**Objective:**

This study examined publicly visible AI adoption among National Cancer Institute (NCI)–designated cancer centers in the United States and assessed whether adoption was associated with institutional characteristics, socioeconomic context, geographic distribution, and state-level political environment.

**Methods:**

We assembled a national dataset of 75 NCI-designated cancer centers using publicly available sources. AI adoption was measured across 3 domains (screening, treatment, and patient care) and summarized as a composite index (0-3). Spatial clustering was evaluated using Moran *I*. Ordered logistic regression models examined associations between AI adoption and institutional factors, including physician workforce size, hospital beds, cancer center type, and contextual factors, including population characteristics, socioeconomic indicators, and state political environment.

**Results:**

Among the 75 cancer centers, the mean AI adoption index was 1.37 (SD 0.86), indicating adoption in approximately 1 to 2 domains on average. Publicly visible AI adoption was most common for screening applications (mean 0.86, SD 0.35), followed by patient care applications (mean 0.50, SD 0.50), and treatment-related applications (mean 0.22, SD 0.42). Moran *I* showed no statistically significant spatial autocorrelation, suggesting that AI adoption did not follow a clear geographic clustering pattern. In regression models, institutional capacity measures, including physician workforce size and hospital bed capacity, showed positive but generally modest associations with AI adoption. State-level socioeconomic indicators, including income, education, and urbanicity, were not consistently associated with adoption. Political-context findings were mixed and should be interpreted cautiously; Republican Party control was associated with higher AI adoption in the primary adjusted model, while the exploratory interaction model suggested that cancer center type and governance context may jointly shape publicly visible AI adoption.

**Conclusions:**

AI adoption among US cancer centers appears uneven across clinical domains and is more closely associated with institutional capacity than with surrounding community socioeconomic characteristics. The absence of statistically significant spatial clustering suggests that, if AI diffusion is occurring, it may be driven primarily through nonspatial channels, such as institutional resources, academic networks, vendor partnerships, or policy environments, rather than geographic proximity alone. Because the study relies on public-source reporting, the findings should be interpreted as patterns of publicly visible AI adoption rather than confirmed clinical integration. Future research should use longitudinal designs, direct institutional validation, and more detailed implementation measures to assess whether AI-enabled cancer care is diffusing equitably across institutions and populations.

## Introduction

### AI Adoption by Cancer Institutions

AI technologies are rapidly transforming cancer screening, treatment, and care by improving early detection, diagnostic accuracy, and personalized treatment planning. These advancements leverage machine learning (ML), deep learning, and advanced image analysis to support clinicians and enhance patient outcomes across various cancer types. For cancer screening, there are a few AI technologies increasingly adopted by health care institutions, including (1) breast cancer: AI systems in mammography have demonstrated superior accuracy compared to human experts, reducing false positives and negatives, and significantly decreasing radiologist workload while maintaining or improving detection rates [1-3]. (2) Lung cancer: AI assists in low-dose computed tomography (CT) and chest x-ray interpretation, increasing nodule detection accuracy, reducing reading time, and enabling risk stratification for personalized screening [4-6]. (3) Colorectal cancer: AI-powered computer-aided detection systems improve polyp and adenoma detection during colonoscopy, supporting early intervention and reducing progression to cancer [7]. (4) Smart biosensors: AI integrated with 2-dimensional material-based biosensors offers portable, cost-effective, and sensitive tools for early cancer biomarker detection [8]. Additionally, AI is increasingly used to enhance diagnosis and prognosis, including image analysis. AI algorithms enable automated tumor segmentation, characterization, and staging across imaging modalities (mammography, CT, magnetic resonance imaging [MRI], and histopathology), supporting more accurate and reproducible diagnoses [1,2,4]. AI predicts tumor properties such as gene mutations and protein expression, aiding in molecular subtyping and prognosis estimation [4,6,9]. Regarding outcome prediction, AI models forecast treatment response, recurrence risk, and survival, informing clinical decision-making and patient counseling [1,6,9]. AI systems also attempt to streamline hospital administration and patient management processes, with the goal of decreasing administrative burdens and operational inefficiencies that often inflate health care costs [10]. Overall, AI technologies are revolutionizing cancer care through a focus on screening accuracy, diagnostic precision, personalized treatment, and cutting costs.

While AI is increasingly adopted by health institutions, there are still a variety of challenges, including (1) validation and generalizability: many AI models require further validation for reproducibility and broad clinical adoption [1,8,11]; (2) workflow integration: effective integration into clinical practice and regulatory compliance remains an ongoing challenge [1,11]; and (3) data and interpretability: ensuring high-quality data, model transparency, and interpretability is essential for safe and effective AI deployment [1]. Recent work on hospital AI adoption has shown that adoption is uneven across US health care organizations and may vary by neighborhood disadvantage, hospital resources, health system affiliation, and accountable care organization participation [12-14]. These findings suggest that AI adoption is not simply a function of technical availability but also reflects organizational capacity, implementation infrastructure, and policy incentives. However, most prior studies have focused on general hospitals or broad health systems rather than National Cancer Institute (NCI)–designated cancer centers, and few have examined state-level political control as a policy-contextual factor. This study extends this literature by examining publicly visible AI adoption across NCI-designated cancer centers while incorporating institutional characteristics, spatial distribution, socioeconomic context, funding, and state political environment.

### Spatial Distribution of AI Adoption by Health Institutions in the United States

AI adoption by health institutions in the United States has been measured by a variety of ways, including (1) binary domain coding approach which categorizes AI presence in specific functional domains such as diagnostics [15]; (2) count-based adoption indices which refer to quantitative measures that track the frequency or number of AI-related activities such as investments, research output, or policy implementations [16,17]; or (3) service-specific tracking via national surveys which typically involves targeted questionnaires to evaluate attitudes, trust, and acceptance among clinicians and patients for particular AI applications [18-20]. Studies have found that institutional adoption of AI in health care has been unevenly distributed, with significant differences based on geography, hospital characteristics, and the socioeconomic status of the communities served [15,21]. Hospitals in urban, wealthier, and system-affiliated areas are much more likely to adopt AI, while those in economically disadvantaged and rural areas lag behind. Regarding geographic and socioeconomic distribution, first, hospitals serving the most economically disadvantaged neighborhoods (the highest Area Deprivation Index [ADI] Q4) are significantly less likely to adopt AI and ML technologies compared to those in the least deprived areas [15,22,23]. This gap is especially pronounced in workforce management and electronic health record applications [15]. Second, states show wide variation in AI adoption rates: for example, New Jersey leads with nearly 49% of hospitals adopting AI, while New Mexico reports 0% adoption [15]. Third, rural and economically disadvantaged areas are underrepresented in AI adoption, suggesting a risk of widening health disparities as AI becomes more integrated into health care [15,21]. Also, hospital characteristics influencing adoption include (1) larger hospitals, those with outpatient surgical departments, private not-for-profit ownership, teaching status, and those that are part of health systems, which are more likely to adopt AI [21,24]; (2) hospitals with a larger market share are also more likely to implement AI by leveraging it for improved financial and operational performance [24]; and (3) affiliation with accountable care organizations explains a notable portion (12%‐25%) of the differences in AI and ML use across hospitals [15]. Other external and internal key factors affecting AI adoption include macroeconomic conditions, the regulatory environment, technological readiness [25], and organizational and individual readiness [25].

Despite AI’s promising benefits, substantial gaps remain in our understanding of AI adoption across cancer institutions, particularly regarding its spatial distribution and the factors influencing its implementation. The lack of comprehensive evaluations regarding how structural, demographic, and geographic variables impact institutional AI adoption and patient outcomes limits our capacity to improve health outcomes and maximize the benefits of AI technology. To address this gap, comprehensive evaluations of AI technology diffusion are required, specifically focusing on disparities related to geography, public policy environment, and institutional capacities.

### Diffusion of Innovations Theory

Innovation diffusion theory explains how technologies or behaviors spread within a social environment over time. The foundational framework, as defined by Everett Rogers, identifies 4 key elements: innovation itself, communication channels, time, and the social system. These elements interact to determine the rate and pattern of adoption of innovations among entities [26]. Factors such as institutional context, infrastructure, supply-side dynamics, and opinion leadership can either facilitate or hinder the diffusion process [27-32]. Diffusion theory is widely used to design interventions that promote the spread of health care practices. Key concepts generally relate to the evidence base and include intervention attributes, demonstration projects, and adaptation to local contexts [27,33]. In industrial and digital contexts, innovation diffusion frameworks help identify drivers and inhibitors of digital transformation, providing actionable insights for managers and policymakers [34]. Innovation diffusion theory can provide an interdisciplinary framework for understanding how AI technologies spread in cancer centers. Its principles are potentially applicable across sectors, and ongoing research continues to refine the theory to address complex, evolving social and technological contexts. This study evaluates the spatial distribution and multilevel determinants of AI adoption across US cancer centers, with a focus on institutional capacity, socioeconomic context, and the state policy environment, including political control.

Despite growing evidence on the clinical promise of AI in oncology, important gaps remain in understanding how AI adoption is distributed across cancer care institutions in the United States. Much of the existing literature focuses on the technical performance of AI tools, model validation, or specific use cases in imaging, diagnostics, treatment planning, and patient monitoring. Less is known about the organizational and contextual factors associated with whether cancer centers publicly report AI adoption across different domains of cancer care. In particular, limited evidence exists on how institutional capacity, cancer center type, state-level socioeconomic conditions, political context, funding, and geographic location are associated with AI adoption among US cancer centers.

Addressing this gap is important because AI implementation is not determined by technical readiness alone. Adoption may depend on institutional resources, workforce capacity, organizational readiness, policy compatibility, external incentives, and local diffusion environments. Cancer centers with greater clinical infrastructure, stronger digital capacity, or more favorable policy and funding environments may be better positioned to become early adopters, while other centers may face implementation barriers even when AI tools are available. Therefore, examining AI adoption at the institutional and geographic levels can provide insight into how emerging technologies diffuse across cancer care systems and where disparities in AI implementation may arise. To address this knowledge gap, this study examined publicly visible AI adoption among US cancer centers across 3 domains: screening, treatment, and patient care. We constructed an AI adoption index using publicly available institutional websites, media reports, and related public sources, and evaluated whether adoption was associated with institutional characteristics, state-level socioeconomic and political context, funding, and geographic distribution. By focusing on organizational-level adoption rather than technical performance alone, this study contributes evidence on the institutional and contextual determinants of AI diffusion in cancer care.

### Study Objectives and Hypotheses

This study evaluated the spatial distribution and institutional, socioeconomic, and policy determinants of AI adoption across US cancer centers. Guided by diffusion of innovations theory, we examined 3 hypotheses. First, we hypothesized that AI adoption would exhibit spatial clustering consistent with regional diffusion processes. Second, we hypothesized that AI adoption would be positively associated with institutional capacity and system readiness, including physician workforce size, hospital bed capacity, and cancer center type, as well as broader state-level socioeconomic and policy context. Third, we hypothesized that state political control would moderate the association between institutional capacity and AI adoption, such that resource-intensive centers would be more likely to adopt AI in policy environments characterized by unified political control.

## Methods

### Data Sources and Collection

All data analyzed in this study are publicly available from institutional and governmental sources. No individual-level or identifiable human data were used. The dataset compiled for this analysis can be reconstructed from the following open-access sources: NCI Cancer Center Directory [35], American Hospital Directory [36], Ballotpedia [37], and the National Conference of State Legislatures [38]. The dataset and code are available upon request from the corresponding author. The study compiled geolocation data for all 75 NCI-designated cancer centers across the United States, which provide advanced cancer care. These centers, chosen for their standardized designation, serve as a consistent basis for assessing clinical services, scientific leadership, and community engagement. Due to their role in research and innovation, NCI centers are positioned to adopt emerging technologies such as AI in cancer screening, treatment, and care. They also make institutional data publicly accessible, supporting comparability across sites. Focusing on these centers captures a key part of the cancer care landscape where AI adoption is most likely to be measurable. While this excludes smaller hospitals, it offers an initial baseline for understanding innovation patterns in well-funded settings and exploring AI expansion to underserved populations. AI adoption was coded using a structured public-source abstraction process based on a standardized coding framework (Table S1 in Multimedia Appendix 1). A graduate research assistant completed the initial coding of publicly visible AI adoption across screening, treatment, and patient care domains using predefined coding criteria. Cancer centers were coded as having adopted AI only when clear publicly available evidence indicated active AI use in cancer screening, treatment, or patient care workflows. Research-only activities, planned initiatives, or general digital health tools without evidence of implementation were not coded as AI adoption. When publicly available information was ambiguous, the graduate research assistant discussed the case with the principal investigator and first author, and final classifications were determined through consensus. The research team subsequently conducted 3 investigator meetings to review ambiguous cases, ensure consistent application of the coding criteria, and finalize all classifications. Because the verification process was not a fully independent duplicate coding process, interrater reliability statistics such as Cohen κ were not calculated. Because the study relied on institutional websites, press releases, media reports, and other publicly available sources, the outcome was defined as publicly visible AI adoption. Absence of public evidence was coded as no publicly visible adoption rather than definitive absence of internal AI use. Website and public-source review was conducted from July 14, 2025, to July 21, 2025, and AI adoption coding reflects information publicly available during this review period.

### Outcome Measures

AI adoption was operationalized as publicly visible evidence of active institutional use of AI in cancer-related screening, treatment, or patient care workflows. Each domain was coded as a binary indicator based on publicly available evidence from institutional websites, press releases, program descriptions, or media reports. A score of 1 was assigned when a cancer center had publicly documented AI use in that domain, and a score of 0 was assigned when no publicly available evidence was identified. Screening AI included applications related to early detection, medical imaging, pathology, genetic testing, or cancer risk assessment. Treatment AI included applications related to treatment planning, radiation optimization, precision oncology, drug selection, or clinical decision support. Patient-care AI included applications related to patient navigation, symptom monitoring, virtual assistants, care coordination, survivorship support, or other patient-facing digital tools. The composite AI adoption index was calculated as the unweighted sum of the 3 binary domain indicators and ranged from 0 to 3, with higher scores indicating publicly visible AI adoption across more domains. No differential weighting was applied because the index was designed to capture the breadth of publicly documented AI adoption rather than the intensity, maturity, or depth of clinical integration. To distinguish AI adoption from AI research activity, we coded AI adoption only when publicly available evidence indicated active institutional use of AI in cancer-related screening, treatment, or patient care workflows. Research-only activity, academic publications, laboratory development, pilot studies without evidence of implementation, general digital health tools, and planned future initiatives were not coded as adoption.

### Key Variables

Institutional-level characteristics include center size (number of beds and physicians) and specialty classification. We also use each cancer center’s most recent year of National Institutes of Health (NIH) administrative funding as a proxy measure of external financial resources. Because NIH funding is publicly available and consistently reported across NCI-designated cancer centers, it was included as a proxy for research-related financial capacity. State-level socioeconomic and political factors were derived from publicly available sources, such as the US Census, American Community Survey, Ballotpedia, and the National Governors Association [39,40]. Our study’s key variables were median household income, rurality, population density, age distribution, racial and ethnic composition, and educational attainment. In our study, the political context was measured using 2 related but distinct variables. Political affiliation refers to the dominant voting behavior or political leaning of the population in a given state (eg, the proportion of votes cast for Democratic vs Republican presidential candidates in recent elections), which captures the broader sociopolitical environment that may influence public attitudes toward technology and health care. State political affiliation was used as a proxy for broader voter sentiment, whereas party control was used as a proxy for the governing policy environment. Because these measures capture different dimensions of political context, they were interpreted separately. Party control was considered the more direct policy-context measure because it reflects institutional control of state government, while political affiliation reflects broader electoral orientation. Because party control more directly reflects the policy and governance context in which institutional implementation decisions occur, it was treated as the primary political-context predictor. State political affiliation was examined as a contextual and sensitivity measure. The correlation between state political affiliation and party control was moderate (*r*=0.638, *P*<.001), indicating that the measures were related but not interchangeable. These variables capture policymaking power that can directly affect funding, regulations, and incentives relevant to AI adoption in health care. NIH funding was included as a proxy for research-related financial capacity and was not intended to directly measure institutional resources available for AI implementation. We included NIH administrative funding as an available proxy for research-related financial capacity, while recognizing that this measure does not fully capture institutional ownership, private revenue, philanthropy, payer mix, or state-level financial support.

### Statistical Methods and Software

To assess the determinants of AI adoption among cancer centers, we first evaluated spatial clustering using the Moran *I* statistic [41,42]. This allowed for testing whether AI adoption exhibited nonrandom spatial patterns. Subsequently, we used the ordered logistic regression model to estimate the association between AI adoption (categorized into ordinal levels of engagement) and explanatory variables, including institutional characteristics and community-level factors [43-45]. This modeling approach accounts for the hierarchical data structure, with institutions nested within states. All statistical analyses were conducted in Stata 19.5 (StataCorp LLC), while spatial mapping and visualization were performed in ArcGIS Pro (Esri) [46].

We estimated a sequence of ordered logistic regression models to evaluate the association between institutional, state-level, and political factors and the AI adoption index. Although cancer centers were located across multiple states, we did not estimate multilevel random-intercept models because the analytic sample included a limited number of institutions per state, making state-level variance components difficult to estimate reliably. Instead, state-level socioeconomic, demographic, and political characteristics were included as contextual covariates in single-level ordered logistic regression models. Model 1 included institutional capacity indicators only, including physicians per 100, beds per 100, and cancer center type. Model 2 added state-level socioeconomic, demographic, and political covariates, including population per 1 million, population density, urbanicity, educational attainment, state-level median household income per US $10,000, racial composition, unemployment rate, state political affiliation, and party control. Model 3 added an exploratory interaction between cancer center type and state party control to assess whether the association between institutional type and AI adoption varied across governance contexts. Model 4 added total funding per US $1 million as a funding-adjusted sensitivity model. The primary inference was based on Model 2; Models 3 and 4 were interpreted as exploratory and sensitivity analyses, respectively. Statistical significance was assessed using 2-sided tests with *P*=.05 as the threshold for statistical significance. Odds ratios (ORs) and 95% CIs were reported for all regression models. Estimates with CIs crossing 1.00 were interpreted as not statistically significant. Findings with *P* values between .05 and .10 were described, when relevant, as suggestive or directional rather than statistically significant. The primary outcome was the AI adoption index, calculated as the unweighted sum of 3 binary indicators for publicly visible AI adoption in screening, treatment, and patient care domains. The index ranged from 0 to 3 and was intended to measure the breadth of AI adoption across domains rather than the intensity, maturity, or clinical depth of implementation. Because the 3 domains differed in prevalence, we also conducted domain-specific logistic regression sensitivity analyses using screening, treatment, and patient-care AI adoption as separate binary outcomes.

The proportional-odds assumption was evaluated using the Brant test of the proportional odds assumption, implemented with Stata’s estat parallel postestimation command. When evidence of nonproportional odds was detected, a generalized ordered logit model was explored as a sensitivity analysis.

### Ethical Considerations

This study used publicly available organizational-level information from institutional websites, governmental sources, and publicly accessible media reports to construct a dataset of US cancer centers. The study did not involve interaction or intervention with human participants, individual-level health records, identifiable private information, biospecimens, or human tissue. Therefore, the present analysis did not meet the definition of human participants research under the US Department of Health and Human Services Common Rule, 45 CFR 46.102, which defines human participants research as involving a living individual about whom an investigator obtains information or biospecimens through intervention or interaction, or obtains, uses, studies, analyzes, or generates identifiable private information or identifiable biospecimens [47]. Because no human participants’ data were used, institutional ethics board review and informed consent were not required. No case or application number is available because no ethics board application was submitted for this organizational-level public-source analysis. The study was conducted in accordance with principles of transparent reporting, responsible use of publicly available information, and the ethical standards described in the Declaration of Helsinki.

## Results

### Descriptive Characteristics

Descriptive statistics for the variables included in the analysis are presented in Table 1. Among the 75 cancer centers analyzed, the mean AI adoption index was 1.37 (SD 0.86), indicating that centers adopted AI in approximately 1 to 2 domains on average. AI adoption varied across functional domains. Screening-related AI was the most common domain (mean 0.86, SD 0.35), followed by patient-care AI (mean 0.50, SD 0.50), while treatment-related AI was least common (mean 0.22, SD 0.42). These findings suggest that AI implementation across cancer centers remains uneven, with greater emphasis on screening and diagnostic applications than on treatment-related uses. Institutional capacity also varied substantially. Centers employed an average of 1174.55 (SD 3124.17) physicians and had an average of 456.45 (SD 494.47) beds. The average state population represented in the sample was approximately 15.1 (SD 12.0) million, with a mean population density of 261.38 (SD 214.12) people per square mile. On average, 81.33% (SD 12.9%) of the population lived in urban areas, 33% (SD 4.76%) had a college degree, the median household income was US $63,643.97, the White population share (White alone, not Hispanic or Latino) was 71.01% (SD 11.14%), and the unemployment rate was 4.08% (SD 0.49%). Funding averaged US $0.42 (SD $0.70) million per center. When stratified by state political affiliation, overall AI adoption was similar between centers in Democratic-leaning and Republican-leaning states (mean AI adoption index 1.40, SD 0.89 vs 1.32, SD 0.80). Screening AI adoption was slightly higher in Democratic-leaning states (mean AI adoption index 0.88, SD 0.32 vs 0.82, SD 0.39), whereas treatment AI adoption was higher in Republican-leaning states (mean AI adoption index 0.29, SD 0.46 vs 0.19, SD 0.40). Centers in Democratic-leaning states were located in states with higher average population density, urbanicity, educational attainment, median household income, and funding. Centers in Republican-leaning states were located in states with a higher average White population share and a lower unemployment rate.

**Table 1.** Descriptive characteristics of US cancer centers stratified by state political affiliation^a^.

Variable	Democratic-leaning states, mean (SD)	Republican-leaning states, mean (SD)	Overall, mean (SD)
AI adoption index	1.40 (0.89)	1.32 (0.80)	1.37 (0.86)
Screening AI adoption	0.88 (0.32)	0.82 (0.39)	0.86 (0.35)
Treatment AI adoption	0.19 (0.40)	0.29 (0.46)	0.22 (0.42)
Patient-care AI adoption	0.51 (0.51)	0.47 (0.51)	0.50 (0.50)
Physicians	1185.60 (2907.71)	1153.32 (3566.97)	1174.55 (3124.17)
Beds	454.77 (525.14)	459.68 (439.79)	456.45 (494.47)
Population	16,500,000 (13,000,000)	12,400,000 (9601,000)	15,100,000 (12,000,000)
Population density	301.79 (242.16)	183.80 (114.73)	261.38 (214.12)
Urban population (%)	83.93 (12.06)	76.33 (13.23)	81.33 (12.90)
College degree (%)	34.62 (4.01)	29.90 (4.61)	33.00 (4.76)
Median household income	67,588.67 (9647.95)	56,070.16 (5730.66)	63,643.97 (10,099.53)
White population (%)	68.96 (9.92)	74.96 (12.44)	71.01 (11.14)
Unemployment rate	4.25 (0.45)	3.77 (0.42)	4.08 (0.49)
Funding per US $1 million	0.47 (0.83)	0.33 (0.31)	0.42 (0.70)

a Values are means (SD). Political affiliation was coded using state-level political affiliation. Funding is reported in millions of dollars. Sample sizes vary slightly across variables because of missing data.

### Spatial Distribution of AI Adoption

**Figure 1.**
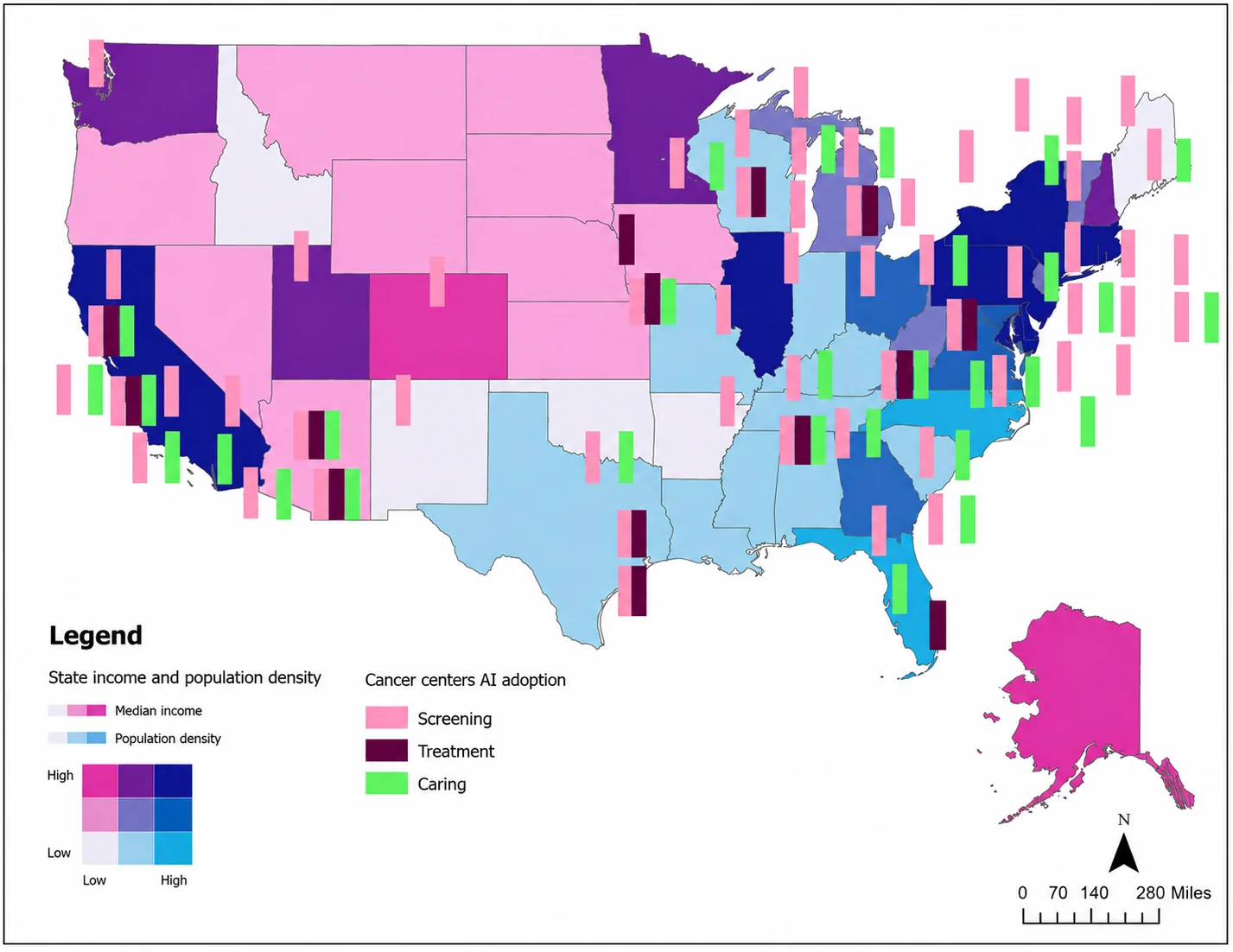
Spatial distribution of AI adoption in US cancer centers: screening, treatment, and care in the context of population density and income.

Figure 1 displays the geographic distribution of publicly visible AI adoption across US cancer centers. Stacked bar symbols indicate whether each center had publicly documented AI use in screening, treatment, and patient care domains, while the background layers show state-level population density and median household income. The map shows descriptive variation in the number and type of AI domains reported across cancer centers. Some centers reported AI adoption across multiple domains, whereas others had publicly visible adoption in one domain or no documented adoption during the review period. However, Moran *I* did not show statistically significant spatial autocorrelation (Moran *I*=−0.0165, *P*=.52). Therefore, the map should be interpreted as a descriptive visualization of geographic variation rather than evidence of statistically significant spatial clustering, regional concentration, or geographic disparity.

To assess potential multicollinearity among the independent variables included in the ordered logistic regression model, we conducted a variance inflation factor (VIF) analysis using ordinary least squares regression. The mean VIF across all predictors was 2.51 (SD 0.94), with individual VIF values ranging from 1.22 to 4.35. None of the variables exceeded the commonly accepted threshold of 10, suggesting that multicollinearity is unlikely to bias the estimation of coefficients in the model. These results indicate that the predictor variables are sufficiently independent to support valid inferences from the regression analysis.

### Regression Analysis

Table 2 presents the ordered logistic regression models examining factors associated with higher AI adoption index scores. Model 1 included institutional capacity indicators only. In this model, physicians per 100 (OR 1.013, 95% CI 0.999‐1.027) and beds per 100 (OR 1.077, 95% CI 0.973‐1.191) were positively associated with higher AI adoption, although neither estimate reached statistical significance. Compared with basic laboratory centers, clinical centers (OR 0.253, 95% CI 0.041‐1.547) and comprehensive centers (OR 0.842, 95% CI 0.195‐3.643) did not differ significantly in Model 1.

**Table 2.** Ordered logistic regression models for AI adoption index among US cancer centers^a^.

Variable	Model 1, OR^b^ (95% CI)	Model 2, OR (95% CI)	Model 3, OR (95% CI)	Model 4, OR (95% CI)
Physicians per 100	1.013 (0.999‐1.027)	1.014 (0.998‐1.030)	1.015 (0.998‐1.031)	1.013 (0.997‐1.030)
Beds per 100	1.077 (0.973‐1.191)	1.098 (0.988‐1.220)	1.101 (0.990‐1.224)	1.096 (0.986‐1.218)
Clinical vs basic laboratory	0.253 (0.041‐1.547)	0.089 (0.010‐0.791)^c^	0.141 (0.005‐3.844)	0.075 (0.007‐0.793^c^)
Comprehensive vs basic laboratory	0.842 (0.195‐3.643)	0.462 (0.089‐2.393)	0.460 (0.064‐3.327)	0.522 (0.087‐3.132)
Population per 1 million	—^d^	1.018 (0.963‐1.077)	1.014 (0.958‐1.073)	1.021 (0.962‐1.083)
Population density	—	0.999 (0.997‐1.002)	0.999 (0.996‐1.002)	0.999 (0.996‐1.002)
Urban population (%)	—	1.007 (0.947‐1.070)	1.008 (0.948‐1.073)	1.003 (0.941‐1.070)
College degree (%)	—	0.982 (0.815‐1.183)	0.961 (0.793‐1.164)	0.976 (0.809‐1.178)
Median income per US $10,000	—	0.934 (0.358‐2.438)	1.034 (0.388‐2.757)	0.957 (0.367‐2.501)
White population (%)	—	0.965 (0.912‐1.022)	0.964 (0.909‐1.023)	0.965 (0.910‐1.023)
Unemployment rate	—	1.115 (0.308‐4.032)	1.126 (0.285‐4.456)	1.187 (0.279‐5.059)
Republican-leaning vs Democratic-leaning	—	0.236 (0.048‐1.165)	0.268 (0.051‐1.401)	0.288 (0.054‐1.527)
Split party control vs Democratic control	—	2.381 (0.347‐16.317)	—	2.556 (0.353‐18.513)
Republican control vs Democratic control	—	13.538 (2.021‐90.680)^e^	—	13.445 (1.965‐92.019)^e^
Basic laboratory x split control	—	—	13.262 (0.246‐715.063)	—
Basic laboratory x Republican control	—	—	2.872 (0.032‐256.924)	—
Clinical x Republican control	—	—	6.730 (0.146‐311.181)	—
Comprehensive x split control	—	—	1.722 (0.210‐14.097)	—
Comprehensive x Republican control	—	—	14.444 (2.101‐99.309^e^)	—
Funding per US $1 million	—	—	—	1.528 (0.798‐2.925)

a Values are odds ratios (95% CI). Model 1 included institutional characteristics. Model 2 added state socioeconomic and political variables. Model 3 added the interaction between cancer center type and party control. Model 4 added total funding per US $1 million as a funding sensitivity analysis. The full dataset included 75 cancer centers. Sample sizes vary across variables and models because of missing covariate, funding, or domain-specific AI adoption data. Model fit was assessed using pseudo *R*2, Akaike information criterion, and Bayesian information criterion. Lower Akaike information criterion and Bayesian information criterion values indicate better fit after accounting for model complexity. Model 2 was treated as the primary adjusted model; Model 3 was exploratory, and Model 4 was a funding-adjusted sensitivity model.

b OR: odds ratio.

c *P*<.05.

d Not applicable.

e *P*<.01.

Model 2 added state-level socioeconomic, demographic, and political covariates and served as the primary adjusted model. The positive associations for physicians per 100 (OR 1.014, 95% CI 0.998‐1.030) and beds per 100 (OR 1.098, 95% CI 0.988‐1.220) remained directionally consistent but were not statistically significant. Clinical centers had significantly lower odds of higher AI adoption compared with basic laboratory centers (OR 0.089, 95% CI 0.010‐0.791), whereas comprehensive centers did not differ significantly from basic laboratory centers (OR 0.462, 95% CI 0.089‐2.393). State-level socioeconomic characteristics, including population size, population density, urbanicity, educational attainment, median income, White population share, and unemployment rate, were not significantly associated with AI adoption. Political context showed a stronger association. Compared with Democratic party control, cancer centers located in Republican-controlled states had higher estimated odds of greater AI adoption (OR 13.538, 95% CI 2.021‐90.680). However, the wide CI indicates substantial uncertainty in the magnitude of this association. In contrast, Republican-leaning state political affiliation was not statistically significantly associated with AI adoption (OR 0.236, 95% CI 0.048‐1.165).

Model 3 examined whether the association between cancer center type and AI adoption varied by state party control. The interaction between comprehensive center status and Republican Party control was statistically significant (OR 14.444, 95% CI 2.101‐99.309). However, several interaction estimates had wide CIs, one interaction cell was empty, and the model included multiple interaction terms. Therefore, Model 3 was interpreted as exploratory rather than as the primary basis for inference.

Model 4 added total funding per US $1 million as a funding-adjusted sensitivity model. The main findings were substantively similar to Model 2. Republican Party control remained significantly associated with higher AI adoption (OR 13.445, 95% CI 1.965‐92.019), and clinical centers remained less likely than basic laboratory centers to have higher AI adoption (OR 0.075, 95% CI 0.007‐0.793). Funding per US $1 million showed a positive but not statistically significant association with AI adoption (OR 1.528, 95% CI 0.798‐2.925). Overall, the funding-adjusted model suggests that the primary findings were not fully explained by available funding capacity.

Table 3 presents the model-fit statistics used to compare the sequential regression models. Model 1, which included institutional capacity indicators only, had a pseudo *R*^2^ of 0.062, Akaike information criterion (AIC) of 186.955, and Bayesian information criterion (BIC) of 203.177. Model 2, which added state-level socioeconomic, demographic, and political covariates, improved model fit based on pseudo *R*^2^ (0.157) and AIC (186.566), although BIC increased to 225.504 because of the larger number of predictors. Model 3, which added exploratory interaction terms between cancer center type and party control, had a pseudo *R*^2^ of 0.168 but higher AIC (190.593) and BIC (236.403), indicating that the added interaction terms did not clearly improve model fit. Model 4, which added funding as a sensitivity analysis, had a pseudo *R*^2^ of 0.162, AIC of 181.569, and BIC of 221.783. Overall, these fit statistics support using Model 2 as the primary adjusted model and interpreting Model 3 as exploratory rather than as the preferred specification. The score test indicated some evidence of departure from the proportional-odds assumption (*χ*²_28_=48.18, *P*=.01). However, attempts to estimate a generalized ordered logit model did not converge because of the modest sample size and model complexity.

**Table 3.** Model fit statistics for ordered logistic regression models of AI adoption.

Variable	Model 1	Model 2	Model 3	Model 4
N	75	73	73	69
Pseudo *R*²	0.062	0.157	0.168	0.162
AIC^a^	186.955	186.566	190.593	181.569
BIC^b^	203.177	225.504	236.403	221.783

a AIC: Akaike information criterion.

b BIC: Bayesian information criterion.

Table 4 presents domain-specific logistic regression sensitivity analyses for screening, treatment, and patient-care AI adoption. In the models without funding adjustment, institutional capacity indicators showed generally positive but nonsignificant associations across domains. Physicians per 100 was positively associated with treatment AI adoption (OR 1.015, 95% CI 0.998‐1.034) and patient-care AI adoption (OR 1.018, 95% CI 0.985‐1.052), although neither association reached statistical significance. Beds per 100 also showed positive but nonsignificant associations across screening (OR 1.088, 95% CI 0.870‐1.361), treatment (OR 1.076, 95% CI 0.955‐1.212), and patient-care AI adoption (OR 1.058, 95% CI 0.938‐1.193). Clinical center status was negatively associated with screening and patient-care AI adoption but positively associated with treatment AI adoption, although estimates were imprecise and not statistically significant. Funding-adjusted domain-specific models showed similar patterns. Funding per US $1 million was not significantly associated with screening AI adoption (OR 0.286, 95% CI 0.030‐2.765) or treatment AI adoption (OR 0.634, 95% CI 0.154‐2.612). However, funding showed a positive and suggestive association with patient-care AI adoption (OR 8.001, 95% CI 0.869‐73.638), although the CI was wide and the estimate did not reach conventional statistical significance. The funding-adjusted patient-care model was statistically significant overall (likelihood ratio [LR] test *P*=.04), whereas the screening model was marginal *(P*=.06) and the treatment model was not significant (*P*=.29). These sensitivity analyses suggest that domain-specific patterns of AI adoption may differ, with funding potentially more relevant to patient-care AI applications than to screening or treatment AI, but the results should be interpreted cautiously because of sparse outcomes, wide CIs, and separation in the treatment models (Table 5).

**Table 4.** Domain-specific logistic regression sensitivity analyses for AI adoption domains^a^.

Variable	Screening, OR^b^ (95% CI)	Treatment, OR (95% CI)	Patient care, OR (95% CI)	Screening with funding, OR (95% CI)	Treatment with funding, OR (95% CI)	Patient care with funding, OR (95% CI)
Physicians per 100	1.004 (0.972‐1.036)	1.015 (0.998‐1.034)	1.018 (0.985‐1.052)	1.006 (0.971‐1.042)	1.015 (0.997‐1.034)	1.015 (0.985‐1.047)
Beds per 100	1.088 (0.870‐1.361)	1.076 (0.955‐1.212)	1.058 (0.938‐1.193)	1.094 (0.870‐1.375)	1.065 (0.944‐1.201)	1.064 (0.940‐1.204)
Clinical vs basic laboratory	0.105 (0.008‐1.465)	2.205 (0.335‐14.525)	0.447 (0.047‐4.217)	0.125 (0.008‐2.014)	2.162 (0.315‐14.854)	0.244 (0.016‐3.800)
Comprehensive vs basic laboratory	1.032 (0.086‐12.421)	—^c^	0.961 (0.169‐5.468)	1.160 (0.091‐14.808)	—	0.801 (0.122‐5.239)
Funding per US $1 million	—	—	—	0.286 (0.030‐2.765)	0.634 (0.154‐2.612)	8.001 (0.869‐73.638)

a Values are odds ratios (95% CI). Domain-specific models used logistic regression with each AI adoption domain coded as a binary outcome. The treatment models showed separation because no basic laboratory centers reported treatment AI adoption. Funding-adjusted models included total funding per US $1 million. The full dataset included 75 cancer centers. Sample sizes vary across variables and models because of missing covariate, funding, or domain-specific AI adoption data.

b OR: odds ratio.

c Not applicable.

**Table 5.** Model fit statistics for domain-specific logistic regression sensitivity analyses of AI adoption.

Variable	Screening	Treatment	Patient care	Screening with funding	Treatment with funding	Patient care with funding
N	67	58	62	63	55	59
Pseudo *R*²	0.156	0.079	0.055	0.196	0.079	0.142
LR^a^ test *P* value	0.07	0.17	0.32	0.06	0.29	0.04

a LR: likelihood ratio.

## Discussion

### Principal Findings

These findings provide mixed support for the study hypotheses and refine how AI diffusion should be interpreted in this setting. Hypothesis 1, which predicted spatial clustering of AI adoption, was not supported. Moran *I* showed no statistically significant spatial autocorrelation (Moran *I*=−0.0165, *P*=.52), indicating that publicly visible AI adoption did not follow a clear geographic clustering pattern. Although the maps showed descriptive geographic variation, these patterns should not be interpreted as evidence of strong regional clustering or spatial diffusion. Instead, the findings suggest that geographic proximity alone may not be the primary mechanism through which AI adoption spreads across cancer centers. AI diffusion may occur through nonspatial channels, including academic networks, national oncology collaborations, NIH funding mechanisms, vendor partnerships, professional conferences, and institutional affiliations. Alternatively, diffusion may remain uneven and incomplete, with adoption shaped more by organizational readiness, workforce capacity, governance context, and public visibility than by geographic proximity. Hypothesis 2, which focused on institutional capacity, was partially supported. Physicians per 100 and beds per 100 were positively associated with AI adoption across models, but these associations were modest and did not reach statistical significance. Hypothesis 3, which focused on the political and governance context, was also partially supported. The political context variables showed different patterns. State political affiliation and party control were moderately correlated (*r*=0.638, *P*<.001), but they captured distinct dimensions of political context. Republican-leaning state political affiliation was directionally associated with lower odds of higher AI adoption, but the CI crossed 1.00, and the association was not statistically significant. In contrast, cancer centers located in Republican-controlled states had higher estimated odds of AI adoption in the primary adjusted model. However, the corresponding CI was wide, indicating substantial statistical uncertainty regarding the magnitude of this association. The exploratory interaction model further suggested that Comprehensive cancer centers in Republican-controlled states had higher odds of greater AI adoption. However, these findings should be interpreted cautiously because political affiliation and party control are correlated, CIs were wide, and the interaction model was exploratory. Overall, the results suggest that publicly visible AI adoption may be more closely related to institutional and policy-contextual factors than to spatial clustering.

The composite AI adoption index should be interpreted as a measure of adoption breadth rather than adoption intensity or clinical maturity. Because screening AI was more commonly reported than treatment or patient-care AI, the index may partly reflect the wider diffusion and greater public visibility of screening-related applications. Domain-specific sensitivity analyses suggested that adoption patterns may differ across AI use cases. Institutional capacity indicators were generally positive but nonsignificant across domains. Funding showed a suggestive positive association with patient-care AI adoption but not with screening or treatment AI. Because NIH funding primarily reflects research capacity rather than the broader financial, operational, or organizational resources required for AI implementation, this finding should be interpreted cautiously.

Guided by diffusion of innovations theory, the findings suggest that variation in publicly visible AI adoption may be associated with institutional resources and the broader policy environment. Specifically, physicians per 100 and beds per 100 showed positive but nonsignificant associations with higher AI adoption across models, but the CI crossed 1.00, and the association was not statistically significant. Therefore, these findings should be interpreted as suggestive patterns rather than statistically significant associations. These results align with previous research indicating that resource-rich hospitals often serve as early adopters of innovation due to greater technical capacity [48-50]. However, the NIH funding measure used in this study should be interpreted as a proxy for research-related financial capacity rather than a direct measure of institutional AI implementation resources. Consequently, any observed associations involving NIH funding should not be interpreted as evidence that research funding alone drives AI adoption. A particularly noteworthy finding is the negative association between being a specialty cancer center and AI adoption. This result may reflect operational rigidity, limited compatibility between AI applications and specialized workflows, or a stronger reliance on conventional expertise. This effect was moderated by political context. Cancer centers located in Republican-controlled states had higher estimated odds of publicly visible AI adoption in the adjusted analysis. However, because the estimated association was accompanied by a wide CI, the magnitude of this relationship remains uncertain and should be interpreted cautiously. Prior studies have supported the idea that political cohesion can influence health care innovation by generating opportunity or stability through shaping administrative processes and funding priorities [51-53]. The association between party control and publicly visible AI adoption should be interpreted as reflecting broader governance and implementation context rather than a direct causal political effect. AI adoption in health care is shaped not only by external regulation but also by institutional governance, organizational readiness, procurement capacity, risk management, workforce preparedness, and implementation infrastructure. From this perspective, unified party control may function as a proxy for a more coordinated policy environment in which health systems face clearer signals about innovation priorities, public-private partnerships, technology investment, and implementation incentives. These mechanisms may amplify institutional capacity, particularly among centers with greater clinical infrastructure and administrative resources. However, political context may also be correlated with unmeasured features of state innovation ecosystems, health care markets, philanthropic networks, or institutional communications capacity. Furthermore, given the relatively small sample of cancer centers included in this study, residual confounding and model instability cannot be ruled out. Therefore, the observed association between party control and AI adoption should be interpreted cautiously as hypothesis-generating evidence that governance context may shape the visibility and diffusion of AI adoption in cancer care.

No significant associations were observed for socioeconomic indicators, including income, education, or urbanicity. This may indicate that AI adoption in cancer care is more closely tied to internal institutional capacity and state-level governance than to external demographic characteristics. Nevertheless, the descriptive maps suggest geographic variation in the distribution of AI-enabled cancer centers. In particular, many rural and underserved regions, especially in western and central Texas, appear to have fewer AI-enabled cancer centers. These descriptive observations should not be interpreted as evidence of statistically significant spatial clustering but may help identify areas for future investigation. This observation is consistent with national reports showing that hospitals serving low-resource areas are significantly less likely to implement AI and ML technologies [15,54-56].

From a public health perspective, these findings are critical. AI has demonstrated potential to improve early cancer detection, tailor treatment regimens, and reduce administrative inefficiencies [5,9,57-63]. Otherwise, regulatory uncertainty, reimbursement challenges, and limited integration with clinical workflows remain factors that tend to limit implementation [64-73]. Without targeted investments and supportive policy environments, there is a risk that existing health disparities will widen as AI becomes increasingly embedded in oncology care.

Collectively, these findings underscore that institutional capacity, particularly the number of physicians and available beds, is the most robust and consistent predictor of AI adoption in cancer centers. However, political context may also contribute to variation in publicly visible AI adoption, although these findings should be interpreted cautiously because of the relatively small sample size and uncertainty surrounding the estimated effects. For example, comprehensive cancer centers located in Republican-controlled states demonstrated significantly higher publicly visible AI adoption, suggesting that political environments may be associated with institutional conditions that facilitate innovation, including support for comprehensive cancer centers or greater policy coherence. In contrast, broader community-level socioeconomic characteristics, including income, education, and urbanicity, were not significantly associated with AI use, indicating that internal institutional resources and political conditions may be more influential than the surrounding community context.

### Limitations

Several limitations should be acknowledged. First, AI adoption was measured using publicly available institutional websites, press releases, media reports, and related public sources. This approach may introduce reporting bias because cancer centers with stronger public relations capacity, more active website maintenance, or greater incentives to publicize innovation may appear to have higher AI adoption. Conversely, centers that use AI internally but do not publicly report these activities may have been misclassified as having no publicly visible adoption. Therefore, the AI adoption index should be interpreted as a measure of publicly visible AI adoption rather than a complete inventory of all internal AI use. Future studies should validate these findings using surveys, interviews, vendor implementation records, electronic health record integration data, or direct institutional reporting. The cross-sectional nature of the data restricts our ability to draw causal inferences regarding the relationship between institutional, policy-political, and community-level factors and AI adoption. Temporal trends and changes in adoption over time could not be assessed. Second, while we used a rigorous coding process to identify AI use through publicly available information, underreporting or inconsistent terminology may have led to measurement error or omitted cases. Third, the AI adoption index, although multidimensional, may not fully capture the depth, quality, or clinical integration of AI technologies at each institution. Although a standardized coding framework, independent review by a graduate research assistant, and multiple investigator consensus meetings were used to improve classification consistency, a formal interrater reliability statistic (eg, Cohen κ) was not calculated. Future studies should incorporate independent duplicate coding with formal reliability assessment to further evaluate reproducibility. Fourth, our analysis is limited to NCI-designated cancer centers, which may not represent broader hospital systems or community-based cancer care facilities. Fifth, although the proportional-odds assumption showed some evidence of violation, alternative generalized ordered logit models failed to converge because of the relatively small sample of cancer centers and the number of model parameters. Accordingly, the ordered logistic regression findings should be interpreted as approximate estimates of the observed associations rather than precise threshold-specific effects. Finally, the study relies on state-level political indicators, which may obscure more localized policy variations or institutional decision-making processes that influence AI uptake. In addition, the association between Republican Party control and AI adoption was accompanied by a wide CI, reflecting substantial statistical uncertainty. Therefore, the observed relationship should be considered exploratory and interpreted cautiously, as residual confounding and model instability due to the relatively small sample of cancer centers cannot be excluded. Future research should incorporate longitudinal designs, richer measures of adoption fidelity, and substate political environments to enhance the understanding of AI diffusion in cancer care. Financial structure may also shape AI adoption across cancer centers. Institutions with greater access to federal research funding, state support, private investment, philanthropy, or industry partnerships may have more resources to support data infrastructure, workforce training, vendor relationships, and AI implementation. Although this study included NIH administrative funding as a proxy for research-related financial capacity, NIH funding primarily reflects research activity and does not directly measure the operational, financial, or organizational resources required for AI implementation in routine clinical care. Therefore, findings related to NIH funding should be interpreted cautiously. In addition, detailed and comparable measures of institutional ownership, operating revenue, payer mix, philanthropic support, and public versus private financing were not consistently available across all centers. Future studies should examine these financial dimensions more directly to determine how resource structure influences the adoption and scale-up of AI-enabled cancer care.

### Conclusion

AI has transformative potential to enhance cancer care by improving diagnostic accuracy, supporting personalized treatment, and easing caregiving burdens. This study finds that AI adoption among cancer-treating institutions appears to be associated with institutional size, hospital type, and broader policy and governance context, rather than surrounding community characteristics. Although formal spatial analyses did not identify statistically significant geographic clustering, the descriptive geographic patterns observed in the maps may still help inform future planning efforts aimed at improving equitable access to AI-enabled cancer care. The findings highlight the potential health benefits of targeted infrastructure support, payment reform, and coordinated policy action to expand AI access in rural and underserved areas so that all patients, regardless of geography or institutional affiliation, benefit from advances in cancer care.

## Supplementary material

10.2196/96231 Multimedia Appendix 1 Coding framework for publicly visible AI adoption across US National Cancer Institute–designated cancer centers.

## Abbreviations

ADI: Area Deprivation Index
AIC: Akaike information criterion
BIC: Bayesian information criterion
CT: computed tomography
LR: likelihood ratio
ML: machine learning
MRI: magnetic resonance imaging
NCI: National Cancer Institute
NIH: National Institutes of Health
OR: odds ratio
VIF: variance inflation factor
