# Supplementary material for: AI Adoption in US Cancer Centers: National Cross-Sectional Study of Institutional and Policy Determinants
Source: J Med Internet Res. 2026 Aug 26;28:e96231. doi: 10.2196/96231 (PMC13509435; doi:10.2196/96231)
Supplement: Supplementary file 1 [file jmir-v28-e96231-s001.docx]

Multimedia Appendix 1

| **Component** | **Coding Rule** |
| --- | --- |
| Unit of analysis | Individual cancer center |
| Data sources | Publicly available cancer center websites and other publicly available institutional information describing AI-related activities |
| AI adoption = Yes (Code = 1) | Clear publicly available evidence that the cancer center had adopted AI for cancer screening, treatment, or care |
| AI adoption = No (Code = 0) | No clear publicly available evidence that the cancer center had adopted AI for cancer screening, treatment, or care |
| Ambiguous information | The graduate research assistant discussed the case with the principal investigator and first author. Final classification was determined through consensus. |
| Quality assurance | The graduate research assistant independently reviewed each cancer center using the standardized coding framework. The research team conducted three investigator meetings to review ambiguous cases, ensure consistent application of the coding criteria, and finalize all classifications through consensus. |

**Table S1**. Coding Framework for Cancer Center AI Adoption.
